# Mid-season real-time estimates of seasonal influenza vaccine effectiveness in persons 65 years and older in register-based surveillance, Stockholm County, Sweden, and Finland, January 2017

**DOI:** 10.2807/1560-7917.ES.2017.22.8.30469

**Published:** 2017-02-23

**Authors:** Maria-Pia Hergens, Ulrike Baum, Mia Brytting, Niina Ikonen, Anu Haveri, Åsa Wiman, Hanna Nohynek, Åke Örtqvist

**Affiliations:** 1 Department of Communicable Disease Control and Prevention, Stockholm County Council, and Karolinska Institutet, Department of Medicine Karolinska Solna, Unit of Infectious Diseases, Stockholm, Sweden; 2 These authors contributed equally to this work; 3 Impact Assessment Unit, Department of Public Health Solutions, National Institute for Health and Welfare, Helsinki, Finland; 4 Unit for laboratory surveillance of viral pathogens and vaccine preventable diseases, Department of Microbiology, The Public Health Agency of Sweden, Solna, Sweden; 5 Viral Infections Unit, Department of Health Security, National Institute for Health and Welfare, Helsinki, Finland; 6 Vaccination Programme Unit, Department of Health Security, National Institute for Health and Welfare, Helsinki, Finland

**Keywords:** Finland, Sweden, viral infections, influenza, influenza virus, surveillance

## Abstract

Systems for register-based monitoring of vaccine effectiveness (VE) against laboratory-confirmed influenza (LCI) in real time were set up in Stockholm County, Sweden, and Finland, before start of the 2016/17 influenza season, using population-based cohort studies. Both in Stockholm and Finland, an early epidemic of influenza A(H3N2) peaked in week 52, 2016. Already during weeks 48 to 50, analyses of influenza VE in persons 65 years and above showed moderately good estimates of around 50%, then rapidly declined by week 2, 2017 to 28% and 32% in Stockholm and Finland, respectively. The sensitivity analyses, where time since vaccination was taken into account, could not demonstrate a clear decline, neither by calendar week nor by time since vaccination. Most (68%) of the samples collected from vaccinated patients belonged to the 3C.2a1 subclade with the additional amino acid substitution T135K in haemagglutinin (64%) or to subclade 3C.2a with the additional haemagglutinin substitutions T131K and R142K (36%). The proportion of samples containing these alterations increased during the studied period. These substitutions may be responsible for viral antigenic change and part of the observed VE drop. Another possible cause is poor vaccine immunogenicity in older persons. Improved influenza vaccines are needed, especially for the elderly.

## Introduction

Systems for register-based monitoring of vaccine effectiveness (VE) against laboratory-confirmed influenza (LCI) in real time were set up in Stockholm County, Sweden, and in Finland, before the start of the 2016/17 influenza season, using population-based cohort studies [1,2]. In both locations, after an initial moderately high VE of about 50%, a rapid and sharp 20% decline in VE was observed. In addition, reports from hospitals and outpatient clinics indicated that a majority of patients with influenza-like illness (ILI) and severe acute respiratory infection (SARI) were elderly people, i.e. those 65 years and above, and that many of them had been vaccinated with the seasonal influenza vaccine (SIV). We therefore wanted to calculate early and mid-season estimates of influenza VE and compare the results between the two populations. The aim was to evaluate VE for LCI in persons 65 years and above, an age group eligible for free SIV.

## Methods

In both Stockholm County, Sweden, with 2 million inhabitants, and Finland with 5.5 million inhabitants, permanent residents have a unique personal identification number (PIN) based on which various national registers can be linked.

In Stockholm County, we used the central database (VAL) for healthcare utilisation, consultations and diagnoses, the vaccination register (Vaccinera) and for the outcome, the national electronic surveillance system (SmiNet) for the reporting of communicable diseases. Data from VAL, Vaccinera and SmiNet were linked using the same PIN (for details on data sources see [1,3,4]). VAL was used for obtaining data on in- and outpatient diagnoses, comorbidities, age and sex as well as the Stockholm Mosaic system. The latter is a proxy for socioeconomic status based on 11 mutually exclusive categories, e.g. living in a low-income urban apartment block, multicultural suburb, affluent inner city, countryside, by which the County (including Stockholm city) can be divided into 120 smaller urban agglomerations [5]. Vaccinera contains all data, starting from 2009, on influenza and pneumococcal vaccination of persons aged 65 years and older or belonging to medical risk groups. Since the SIV programme in Stockholm offers persons 65 years and older vaccination free of charge and registration is mandatory and required for reimbursements to the healthcare provider, it can be assumed that all vaccinated persons in that age group are included in this database. SmiNet includes all diagnoses of influenza A and B starting from 1 December 2015 when they became notifiable diseases.

In Finland, the Population Information System (PIS) [5], the National Vaccination Register (NVR) [6] and the National Infectious Diseases Register (NIDR) [7] are also linked through a unique PIN. The PIS provides information on every person’s date of birth, sex, date of death, and residential history. Also the NVR contains individual-level data, e.g. vaccine type and lot number as well as date of vaccination, for all vaccinations given within public primary healthcare (the system responsible for delivering the national immunisation programme), including free SIV for certain age and risk groups. The coverage of the NVR is assumed to reach 100% when excluding the population (< 5% of the elderly) that is affected by identified regional and temporal gaps in the NVR [6] or was temporarily living abroad during the study period. As part of the National Notification System of Communicable Diseases mandated by the Communicable Disease Act [8], all laboratories must send to the NIDR individual-level data on respiratory specimens that test positive for influenza, e.g. influenza type, date and place of sampling. The samples are taken on clinical grounds by judgement of the treating physician both in inpatient and outpatient settings.

The study populations were formed by the elderly, i.e. all individuals aged 65 years and older registered in Stockholm County on 1 October 2016 and all individuals aged 65 to 100 years permanently living in Finland on 1 October 2016. 

The vaccines used for adult SIV during the current season in Stockholm were Vaxigrip (Sanofi Pasteur MSD, Lyon, France) (94.7%) and Fluarix (GSK, Brentford, United Kingdom) (5.2%), County. In Finland, it was Influvac (Abbot, Illinois, United States) in public healthcare and Vaxigrip in private healthcare. An individual was defined as vaccinated (exposed) starting from the day after (first) SIV during the ongoing season, and as previously vaccinated if they had at least one SIV record in the respective vaccination register for the previous 2015/16 season.

The outcome was defined as any LCI, irrespective of the influenza (sub)type, in patients sampled as in- or outpatients anywhere in the healthcare system.

### Statistical analyses

Hazard rate ratios (HRR) comparing the hazard rates of LCI among vaccinated and unvaccinated individuals were calculated using Cox regression analyses. Vaccination status was modelled as a time-varying exposure, so individuals could contribute both vaccinated and unvaccinated risk time. The follow-up time, that the individuals of the two study populations contributed to started on 1 October 2016 and ended with the occurrence of LCI, death (Finland only), or on 15 January 2017 (end of week 2), whatever occurred first. The cut-off in the data on 15 January reflects the time point when this publication was prepared. VE was calculated as (1 – adjusted HRR) × 100% and reported with 95% confidence intervals (CI).

The Cox models were adjusted for age in years (65–69, 70–74, 75–79, 80–84, ≥ 85 or 85–100), sex, previous influenza vaccination, and in Stockholm County also for comorbidity status, socioeconomic status and pneumococcal vaccination. The potential of previous influenza vaccination being an effect modifier was evaluated by stratifying the analysis and comparing the hazard rates among people vaccinated neither in 2015/16 nor in 2016/17 and among people vaccinated in both seasons, people vaccinated only in 2015/16 and people vaccinated only in 2016/17.

In sensitivity analyses, time since vaccination was taken into account and the time-dependent exposure variable was modified. Instead of only two levels (‘not vaccinated’, ‘vaccinated for 1 day or more’), three levels (‘not vaccinated’, ‘vaccinated for 1 to 14 days’, and ‘vaccinated for 15 days or more’) and seven levels (‘not vaccinated’, ‘vaccinated for 1 to 7 days’, ‘vaccinated for 8 to 14 days’, ‘vaccinated for 15 to 29 days’, ‘vaccinated for 30 to 44 days’, ‘vaccinated for 45 to 89 days’, and ‘vaccinated for 90 days or more’) were considered and the respective VE estimates calculated.

In addition, the analyses were stratified by age calculating separate VE for the study population younger than 75 years and the study population aged 75 years and older.

Data management and analyses on the Swedish side were carried out using SAS Enterprise software (SAS Institute Inc., Cary, NC) and R 3.3.2 on the Finnish side.

### Virus characterisation

A subset of influenza-positive specimens from clinical laboratories and sentinel surveillance systems, including patients treated in intensive care units (ICU), was chosen and characterised by sequencing of the haemagglutinin gene.

The chosen Finnish and Swedish strains represented different geographic origins and were timely distributed between weeks 40/2016 and 2/2017. Of the 158 sequenced samples, 43 of 75 (57%) and 34 of 83 (41%) were from the Finnish and Swedish sentinel systems, respectively. The remaining sequenced samples were from several clinical laboratories in both countries during the studied period.

### Ethical consideration

The analysis in Stockholm was part of an ongoing evaluation of vaccine programmes required by the Department of Communicable Disease Control and Prevention, Stockholm County Council, Stockholm, Sweden, and falls outside the mandate for the Regional Ethics committee. PINs were anonymised in the linking of Vaccinera to VAL and SmiNet, and no data making individual identification possible was retained.

The National Institute for Health and Welfare, Finland (THL) carries out IVE evaluations as its statutory duty mandated by the Communicable Disease Act [6]. The umbrella protocol for influenza studies in context of the national immunisation programme, including the analyses presented here, have been reviewed by the THL Ethical committee and by the data ombudsman of Finland (THL/607/6.02.00/2016).

## Results

**Figure 1:**
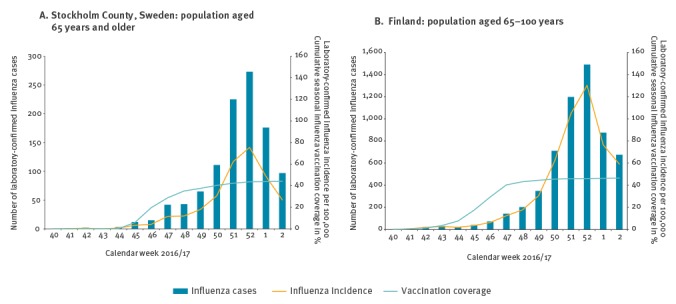
Coverage of seasonal influenza vaccination and number and incidence of laboratory-confirmed influenza cases, by calendar week, Stockholm and Finland, 1 October 2016–15 January 2017 (n = 358,583 and 1,144,894, respectively)

The 2016/17 influenza epidemic started earlier than usual both in Sweden and Finland (Figure 1). The first cases were seen already in early November and the epidemic peaked in week 52. In both countries influenza A dominated (> 99%). Nearly all samples were influenza A(H3N2); only 10 of more than 1,300 typed samples in Sweden were influenza A(H1N1). In Finland, almost 17,000 laboratory-confirmed influenza A findings were reported to NIDR during the follow-up period. The National Influenza Centre in Finland subtyped a total 122 samples, and all were influenza A(H3N2).

In total, 1,034 and 5,845 LCI cases aged 65 years or above were reported during the study period in Stockholm County and Finland, respectively. The baseline characteristics of the population are presented in Table 1.

**Table 1:** Comparison of baseline characteristics in the study population of Stockholm and Finland, 1 October 2016–15 January 2017 (n = 358,583 and 1,144,894, respectively)

	Not vaccinated		Vaccinated	
	n	%	n	%
**Stockholm County, Sweden**	**n = 201,106**		**n = 157,477**	
**Age group**				
65–69 years	71,999	36	35,128	22
70–74 years	54,972	27	46,418	29
75–79 years	30,787	15	32,158	20
80–84 years	19,817	10	21,572	14
≥ 85 years	23,531	12	22,201	14
**Sex**				
Male	91,184	45	69,482	44
Female	109,922	55	87,995	56
**Previous influenza vaccination**				
Not vaccinated in 2015/16	155,831	77	38,790	25
Vaccinated in 2015/16	45,275	23	118,687	75
**Finland^a^**	**n = 612,818**		**n = 532,076**	
**Age group**				
65–69 years	219,447	36	157,586	30
70–74 years	136,560	22	134,782	25
75–79 years	99,974	16	108,800	20
80–84 years	72,647	12	72,593	14
85–100 years	84,190	14	58,315	11
**Sex**				
Male	263,972	43	234,226	44
Female	348,846	57	297,850	56
**Previous influenza vaccination**				
Not vaccinated in 2015/16	535,248	87	125,622	24
Vaccinated in 2015/16	77,570	13	406,454	76

^a^ By vaccination status as of 15 January 2017, i.e. follow-up was not restricted after a person was diagnosed with laboratory-confirmed influenza.

In Stockholm, 97% of the individuals with LCI had been sampled in the hospital setting, either in the emergency room or on a ward. Of the 1,034 patients with LCI, 755 (73%) were treated as inpatients. In Finland, no less than 3,787 (65%) of patients with LCI had been sampled in the hospital setting (when considering all places of sampling not unambiguously identifiable as outpatient), but no information about the setting of further treatment, i.e. whether the patient was transferred to a ward or sent home, was available for the present analysis.

The SIV campaign in Stockholm County started on 9 November 2016 (week 45). By 30 November, 100,442 persons 65 years and older were vaccinated, which corresponded to 28% of this age group, and by 31 December, the corresponding figure was 152,583 (43%) (Figure 1). In Finland, the SIV campaign started gradually, and most of the vaccinations were given in weeks 45–47. By the end of week 47, 461,323 (40%) of the 1,144,894 elderly people included in the study were vaccinated. The SIV coverage further increased to 46% by the end of 2016 (Figure 1).

A stratified analysis demonstrated (data not shown) that previous SIV (Table 1) was not an effect modifier, neither in the Stockholm nor in the Finnish data.

**Figure 2:**
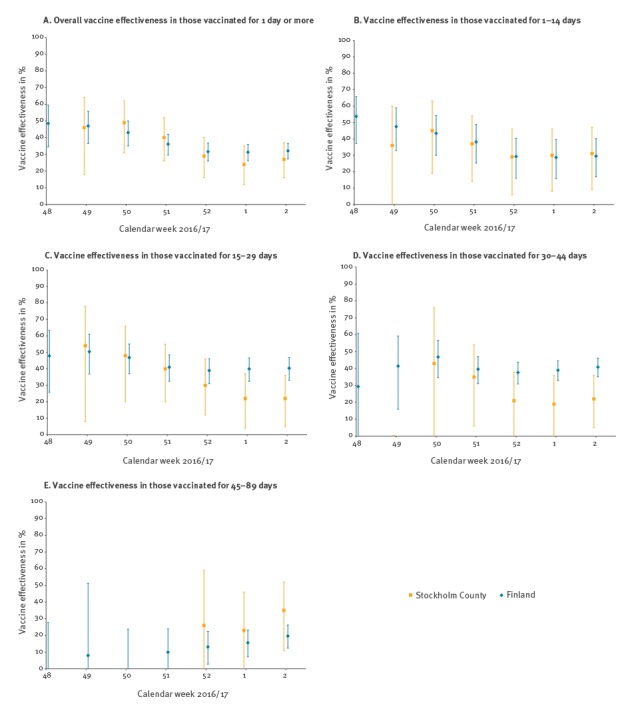
Weekly estimates of influenza vaccine effectiveness in the population aged 65 years and older in Stockholm County^a^, Sweden and 65–100 years in Finland^b^, 1 October 2016–15 January 2017 (n = 358,583 and 1,144,894, respectively)*

In Stockholm, the first two estimates of VE, in weeks 49 and 50, were 56% (95% CI: 11–78) and 49% (95% CI: 38–70) (Figure 2). After that, VE declined rapidly to the current estimate of 28% (95% CI: 16–37) in week 2, 2017 (Figure 2, Table 2). In Finland, the VE in weeks 48 and 49 was estimated at 49% (95% CI: 34–60%) and 47% (95% CI: 36–56%) (Figure 2). In the following weeks, VE dropped to the current (week 2) estimate of 32% (95% CI: 27–37) (Figure 2, Table 2). There was no significant difference when comparing VE of individuals considered vaccinated from day 1 after vaccination or considered vaccinated from day 15 after vaccination, with the unvaccinated as a reference (Table 2).

Panel A: whole population, unstratified. Panels B–E: whole population, stratified according to time being vaccinated.

^a^ Models were adjusted for age, sex, comorbidity status, socioeconomic status, previous seasonal vaccination and pneumococcal vaccination. As complete case analysis was used, the number of cases decreased due to missing data on socioeconomic status.

^b^ Models were adjusted for age, sex and previous seasonal vaccination.

**Table 2:** Vaccine effectiveness estimates for seasonal influenza vaccination on laboratory-confirmed influenza in persons 65 years and older, Stockholm and Finland, 1 October 2016–15 January 2017 (n =358,583 and 1,144,894, respectively)

	Cases	Person-years	Population^a^	Crude hazard rate ratio (95% CI)	Adjusted hazard rate ratio (95% CI)	Vaccine effectivene % (95% CI)
**Stockholm County, Sweden^b^**						
Unvaccinated	654	83,263	201,113	Ref	Ref	Ref
Vaccinated for 1 day or more^c^	380	20,736	157,470	0.90 (0.79–1.03)	0.72 (0.63–0.89)	28 (16–37)
Vaccinated for 15 days or more	322	14,345	153,762	0.94 (0.82–1.08)	0.76 (0.65–0.89)	24 (11–35)
**Finland^d^**						
Unvaccinated	3,674^e^	247,456	613,202	Ref	Ref	Ref
Vaccinated for 1 day or more^c^	2,171	85,674	531,692	0.73 (070–0.77)	0.68 (0.64–0.73)	32 (27–37)
Vaccinated for 15 days or more	2,006	65,357	527,664	0.73 (0.70–0.78)	0.67 (0.63–0.72)	33 (28–38)

CI: confidence interval.

^a^ By vaccination status at the end of each individual’s follow-up.

^b^ Models were adjusted for age, sex, comorbidity status, socioeconomic status, previous seasonal vaccination and pneumococcal vaccination. As complete case analysis was used, the number of cases decreased due to missing data on socioeconomic status.

^c^ The sensitivity analysis showed that there was no protection during the first week after vaccination, but that a significant vaccine effectiveness could be observed already during days 8 to 14 (see text).

^d^ Models were adjusted for age, sex and previous seasonal vaccination.

^e^ The number of vaccinated/unvaccinated differs from Table 1 because 384 people were vaccinated after having a laboratory-confirmed influenza. For Table 2, they are counted as unvaccinated and then their follow-up was stopped because they turned out to be a case.

A sensitivity analysis revealed that VE for ‘being vaccinated for 1 to 7 days’ was < 0% (95% CI: < 0–15%) in Stockholm and 17% (95% CI: −5 to 35%) in Finland, while VE for ‘being vaccinated for 8 to 14 days’ was 30% (95% CI: 4–49) and 37% (95% CI: 22–49) in Stockholm and Finland, respectively. VE for a later time after vaccination seemed more or less stable during the study period (Table 3, Figure 2 panels C and D). In Finland, VE estimates started to decrease when the exposure to SIV was 45 days or more in the past (Table 3). However, an exact evaluation of the onset of declining VE was not done. The Stockholm VE estimates were generally lower and characterised by broad confidence intervals because of small case numbers. In Stockholm, but not in Finland, the VE in persons older than 75 years were much lower than those aged 65–74 years (data not shown).

**Table 3:** Vaccine effectiveness estimates for seasonal influenza vaccination on laboratory-confirmed influenza in persons 65 years and older, by time since vaccination, Stockholm and Finland, 1 October 2016–15 January 2017 (n = 358,583 and 1,144,894, respectively)

	Cases	Person-years	Crude hazard rate ratio (95% CI)	Adjusted hazard rate ratio (95% CI)	Vaccine effectiveness % (95% CI)
**Stockholm County, Sweden^a^**					
Unvaccinated	654	83,263	Ref	Ref	Ref
Vaccinated for 1–14 days^b^	58	5,960	0.84 (0.65–1.17)	0.69 (0.53–0.91)	31 (9–47)
Vaccinated for 15–29 days	132	6,167	0.96 (0.79–1.23)	0.78 (0.64–0.95)	22 (5–36)
Vaccinated for 30–44 days	130	5,149	0.97 (0.80–1.17)	0.78 (0.64–0.95)	22 (5–36)
Vaccinated for 45–89 days	59	3,027	0.79 (0.58–1.07)	0.65 (0.48–0.89)	35 (11–52)
Vaccinated for 90 days or more	1	1	NA	NA	NA
**Finland^c^**					
Unvaccinated	3,674	247,456	Ref	Ref	Ref
Vaccinated for 1–14 days^b^	165	20,317	0.74 (0.63–0.87)	0.71 (0.60–0.83)	30 (17–40)
Vaccinated for 15–29 days	369	21,529	0.63 (0.56–0.70)	0.60 (0.53–0.67)	40 (33–47)
Vaccinated for 30–44 days	675	20,641	0.63 (0.58–0.69)	0.59 (0.54–0.65)	41 (35–46)
Vaccinated for 45–89 days	957	23,107	0.89 (0.83–0.96)	0.80 (0.74–0.88)	20 (12–26)
Vaccinated for 90 days or more	5	80	2.11 (0.87–5.08)	1.69 (0.70–4.08)	−69 (−308 to 30)

CI: confidence interval; NA: not applicable.

^a^ Models were adjusted for age, sex, comorbidity status, socioeconomic status, previous seasonal vaccination and pneumococcal vaccination. As complete case analysis was used, the number of cases decreased due to missing data on socioeconomic status.

^b^ The sensitivity analysis showed that there was no protection during the first week after vaccination, but that a significant vaccine effectiveness could be observed already during days 8 to 14 (see text).

^c^ Models were adjusted for age, sex and previous seasonal vaccination.

### Genetic analyses

**Figure 3:**
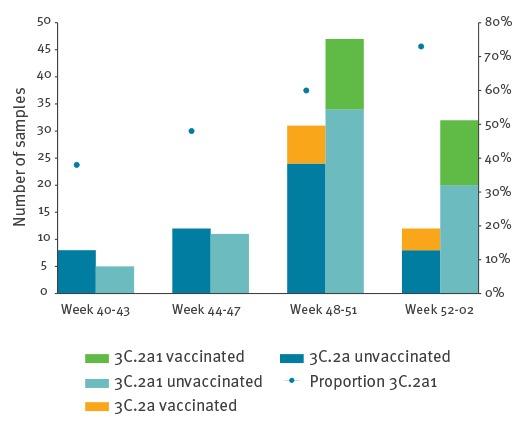
Subclade distribution of influenza A(H3N2) viruses from unvaccinated and vaccinated patients, Stockholm and Finland, 1 October 2016–15 January 2017 (n = 158)

**Figure 4:**
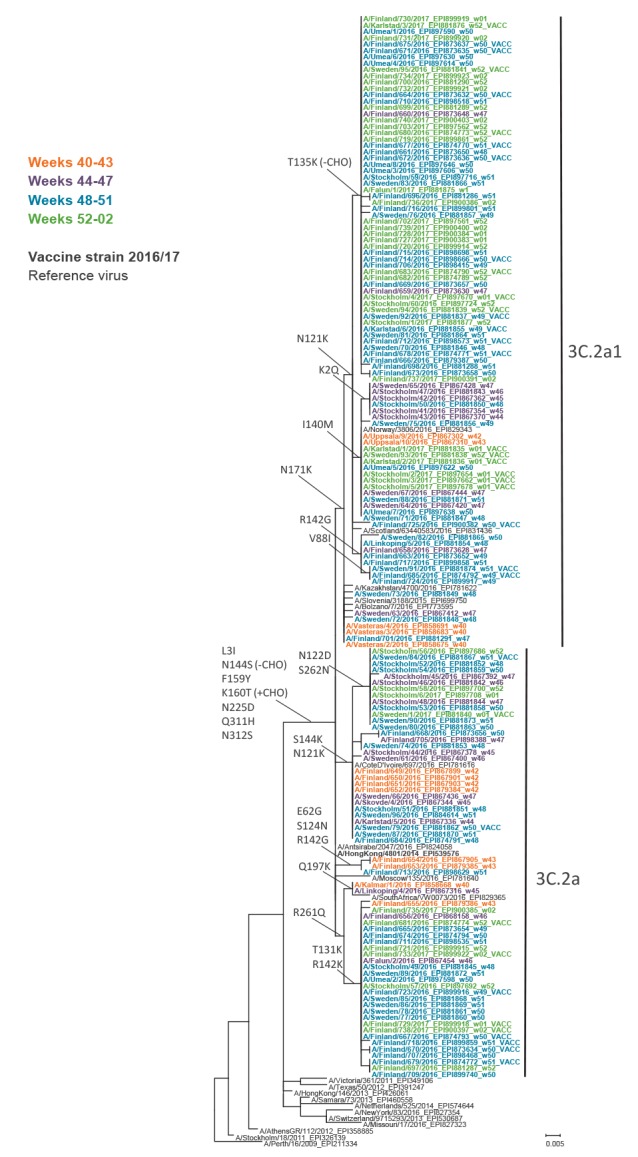
Phylogenetic analysis of amino acid sequences of the haemagglutinin HA1 subunit in influenza viruses from patients in Sweden and Finland, 1 October 2016–15 January 2017

Characterisation of influenza A(H3N2) samples from Sweden and Finland showed that all viruses belonged to subclades 3C.2a or 3C.2a1, which are both considered to be antigenically similar to the vaccine strain A/Hong Kong/4801/2014 [7]. In total 158 influenza A(H3N2) viruses were sequenced, 121 from unvaccinated and 37 from vaccinated patients. The proportion of viruses belonging to subclade 3C.2a1 (n = 95) increased during the study period from 38% to 73% (Figures 3 and 4). In addition, the amino acid substitutions T135K and G479E in the HA1 and HA2 part of the haemagglutinin were determined in 58 of the 95 subclade 3C.2a1 viruses (Figure 4). Twenty-five of the 95 3C.2a1 viruses and 16 of the 58 viruses with the T135K and G479E substitutions were from vaccinated patients. Among the 63 viruses in subclade 3C.2a, 12 were samples from vaccinated persons. Nine of these 12 samples had the additional amino acid substitutions T131K, R142K and R261Q in HA1. All sequences have been uploaded to the Global Initiative on Sharing All Influenza Data (GISAID) EpiFlu database (Figure 4). Table 4 lists all reference sequences retrieved from GISAID for the phylogenetic analysis. The authors gratefully acknowledge the originating and submitting laboratories who contributed sequences were used in this study.

Dots: proportion of influenza subclade 3C.2a1 among total characterised samples, irrespective of vaccination status.

The strains are coloured according to collection period, and samples from vaccinated patients have VACC as a suffix after the week number. The tree was constructed using the maximum likelihood method with Mega software version 5.1. The reference sequences (black colour) were downloaded from the Global Initiative on Sharing Avian Influenza Data (GISAID) and listed in Table 4.

**Table 4:** Details of the influenza A(H3N2) reference sequences retrieved from the Global Initiative on Sharing Avian Influenza Data (GISAID)’s EpiFlu database for phylogenetic analysis of HA1 in this study

Isolate name	Segment ID	Country	Originating laboratory	Submitting laboratory
A/Perth/16/2009	EPI211334	Australia	WHO Collaborating Centre for Reference and Research on Influenza	Centers for Disease Control and Prevention
A/Stockholm/18/2011	EPI326139	Sweden	Swedish Institute for Infectious Disease Control	National Institute for Medical Research
A/AthensGR/112/2012	EPI358885	Greece	Hellenic Pasteur Institute	National Institute for Medical Research
A/Missouri/17/2016	EPI827323	United States	Missouri Department. of Health & Senior Services	Centers for Disease Control and Prevention
A/Switzerland/9715293/2013	EPI530687	Switzerland	Hopital Cantonal Universitaire de Geneves	National Institute for Medical Research
A/New York/83/2016	EPI827354	United States	New York State Department of Health	Centers for Disease Control and Prevention
A/Netherlands/525/2014	EPI574644	The Netherlands	National Institute for Public Health and the Environment (RIVM)	National Institute for Medical Research
A/Samara/73/2013	EPI460558	Russian Federation	WHO National Influenza Centre Russian Federation	National Institute for Medical Research
A/HongKong/146/2013	EPI426061	Hong Kong (SAR)	Government Virus Unit	National Institute for Medical Research
A/Texas/50/2012	EPI391247	United States	Texas Department of State Health Services-Laboratory Services	Centers for Disease Control and Prevention
A/Victoria/361/2011	EPI349106	Australia	Melbourne Pathology	WHO Collaborating Centre for Reference and Research on Influenza
A/South Africa/VW0073/2016	EPI829365	South Africa	Sandringham, National Institute for Communicable D	Crick Worldwide Influenza Centre
A/Moscow/135/2016	EPI781640	Russian Federation	Ivanovsky Research Institute of Virology RAMS	Crick Worldwide Influenza Centre
A/HongKong/4801/2014	EPI539576	Hong Kong (SAR)	Government Virus Unit	National Institute for Medical Research
A/Antsirabe/2047/2016	EPI824058	Madagascar	Institut Pasteur de Madagascar	Crick Worldwide Influenza Centre
A/CoteD'Ivoire/697/2016	EPI781616	Cote d'Ivoire	Pasteur Institut of Côte d'Ivoire	Crick Worldwide Influenza Centre
A/Bolzano/7/2016	EPI773595	Italy	Istituto Superiore di Sanità	Crick Worldwide Influenza Centre
A/Slovenia/3188/2015	EPI699750	Slovenia	Laboratory for Virology, National Institute of Public Health	Crick Worldwide Influenza Centre
A/Kazakhstan/4700/2016	EPI781622	Kazakhstan	National Reference Laboratory	Crick Worldwide Influenza Centre
A/Scotland/63440583/2016	EPI831436	United Kingdom	Gart Naval General Hospital	Microbiology Services Colindale, Public Health England
A/Norway/3806/2016	EPI829343	Norway	WHO National Influenza Centre	Crick Worldwide Influenza Centre

The authors gratefully acknowledge the originating and submitting laboratories who contributed sequences that were used in this study.

## Discussion

Annual vaccination against circulating influenza viruses remains the best strategy for preventing influenza illness. However, VE varies widely and in some seasons the protection of especially older persons and other medical risk groups may be very low or even non-existing, particularly in seasons dominated by influenza A(H3N2) [1,8]. In addition, VE estimates in a given season may differ depending on whether the analysis is performed early/mid-season or at the end of the season, in most cases resulting in lower end-of-season estimates [8,9]. A decrease in VE observed within a season may be due to a change in the circulating virus such as the introduction of a new clade of influenza A(H3N2) during the 2014/15 season [10], to the egg-adaptation of the vaccine influenza A(H3N2) strain [11], or to waning immunity over time [12,13]. Rapid feedback on the impact of SIV is therefore important, as it may help guide the outbreak response.

In our current study, both Stockholm and Finland noted moderately high VE of ca 50% against LCI (predominantly influenza A(H3N2)) in persons 65 years and older, 4 to 5 weeks after the start of the epidemic at the beginning of November which coincided with the start of the SIV campaigns in both countries. However, during the following four weeks, VE declined steeply and was only around 30% by week 2, 2017, in both Stockholm and Finland. Since then and up to week 6, VE has remained stable in both places (data not shown). While the reasons for this observation remain unknown, it seems unlikely that the early decline occurred because the vaccination campaigns were started too late, since the highest VEs were observed early in the season. The sensitivity analysis showed that although there was no protection during the first week after vaccination, a significant VE was observed already during days 8 to 14. Thus, VE was similar irrespective of whether we considered events and person-time accumulating during the first 14 days after vaccination as vaccinated, or whether we excluded them from the analysis. A majority of the study population had been vaccinated also in the previous year and they may therefore have had a rapid immune response to SIV and have been protected earlier than 14 days after vaccination. We believe that it is more correct to either consider persons vaccinated from day 1 or 8 after vaccination or exclude them from the analysis, than to include them in the non-vaccinated group, which results in misclassification biasing the VE estimates towards zero.

During the study period, the proportion of samples in subclade 3C.2a1 increased and in total 60% (95/158) of the viruses were in this subclade. The majority, 25/37 (68%), of the genetically characterised samples from vaccinated patients belonged to subclade 3C.2a1 and 16 of those had the additional amino acid substitution T135K. This mutation is located in a conserved element of the receptor-binding site in the antigenic epitope A and causes a loss of the glycosylation motif [14]. Amino acid 135 is conserved in 62% of all human H1, H2 and H3 viruses [15]. In total 12/63 (19%) of the viruses in subclade 3C.2a were samples from vaccinated persons and nine of these had the additional amino acid substitutions T131K, R142K and R261Q. Both the T131K and the R142K substitution are located in the antigenic epitope A and T131 is conserved in 45% of all human H1, H2 and H3 viruses [15]. In a study from Canada which included all age groups and reported a higher adjusted interim VE of 42% for 2016/17, 80% of the characterised influenza A(H3N2) samples belonged to subclade 3C2a1 and 19% to 3C.2a [16]. Only 19% of the 3C.2a1 samples had the T135K mutation and 74% of the 3C.2a samples had the T131K mutation. In total 29% of the characterised samples in Canada had T135K or T131K, while in our study, one of these two alterations was detected in 54% of all samples, and in 68% of the samples collected from vaccinated patients. The characterised samples were not randomly selected to be representative for vaccinated and unvaccinated persons or different time periods, and it remains to be investigated whether these specific substitutions alter the antigen similarity to the influenza A(H3N2) vaccine strain A/Hong Kong/4801/2014.

A study by Kissling et al. [8] about pooled-season VE against influenza A(H3N2) in persons 60 years and older during the seasons 2011/12 to 2014/15, showed that VE reached a peak of 44.6% at day 45 after vaccination and then gradually declined to 0% at day 140. In contrast, we found a very early VE peak of around 50% and then a rapid decline to a fairly stable low level of around 30% during the remaining study period, which for most individuals was less than 60 days after vaccination, and also during the four weeks after the end of the study period. 

The sensitivity analyses, where time since vaccination was taken into account, could not demonstrate a clear gradual decline, neither by calendar week nor by time since vaccination. We do not have a satisfactory explanation for these observations; the antigenic change, discussed above, or a chance finding are two of several possibilities. As more LCI cases are observed, the power of the study increases and the estimates will become more accurate. Since the confidence intervals of the weekly overall VE estimates are now overlapping, the observed decline is not statistically significant. We will revisit this issue with more cases and follow-up time in the end-season analysis.

However, the generally low VE is probably at least partly a result of the poor immunogenicity of the present SIV in older persons, especially for influenza A(H3N2) [17]. Increased use of adjuvanted vaccines and the introduction of high-dose vaccines in Europe should therefore be considered [18]. In a large randomised placebo-controlled study of persons 65 years of age and older, a high-dose influenza vaccine was 24% more efficacious in the prevention of influenza compared with a standard trivalent vaccine (TIV) [19]. Similarly, in a randomised study in persons 65 years and older, influenza vaccine adjuvanted with MF-59 induced significantly higher antibody response, especially against influenza A(H3N2), than ordinary TIV [20].

A major limitation of this study was the inability to control for healthcare-seeking behaviour and sampling biases. If vaccinated persons with ILI seek healthcare more often and are more likely to be swabbed by doctors than unvaccinated persons, this would underestimate VE, and vice versa. However, we believe this risk to be low in this older age group of patients, most of whom had signs of severe influenza, because nearly all patients in Stockholm and a large part of the patients in Finland were sampled in the hospital setting and treated as inpatients. Elderly persons who are ill enough to seek hospital care because of an infection will do that irrespective of their vaccination status. In the hospital setting, sampling for influenza is performed not only in order to establish a diagnosis and determine the correct treatment, but also because an LCI will mean that the patient can be treated in a cohort with other influenza patients which can prevent the spread of influenza in the hospital. In order to fully understand the dynamics of VE during an influenza season, a more detailed cohort study addressing the potential sources of bias is warranted. Also, a small amount of vaccine exposure misclassification cannot be fully excluded. The Finnish NVR does not cover vaccinations given in the private sector and the number of SIV doses missed by the NVR remains unknown, although it is expected to be a negligible number compared with the ones registered.

The strength of our study is that by using the same PIN to link vaccination, laboratory and diagnostic registers, we could study VE in real time for all inhabitants in two large geographic areas, and that the large number of LCI cases made it possible to perform and communicate an early estimate of VE. The fact that the two sites detected the same signal at the same time and that the evolution of the VE over time followed the same pattern at both sites lends further credibility to our results.

Irrespective of the cause, the low VE in older persons in this interim estimate has had implications for healthcare in Stockholm County and Finland: early antiviral therapy was recommended for ILI and SARI in risk groups, irrespective of vaccine status. Also, to keep the momentum for SIV compliance and provide a better fit, the World Health Organization (WHO) vaccine strain selection committee needs timely evidence for their decision on the composition of the vaccine for the following influenza season. Finally, our study indicates that it is possible to deliver real-time VE estimates by population-based register linkage, although further methodological analyses are needed to understand the potential confounders.

## *Author’s correction:

The x-axes in Figure 2 were mistakenly marked 2015/16 instead of 2016/17. This mistake was corrected on 1 March 2017 on request of the authors.
