# Authors’ correction for Euro Surveill. 2017;22(8)

**DOI:** 10.2807/1560-7917.ES.2017.22.9.30475

**Published:** 2017-03-02

**Authors:** Eurosurveillance editorial team

**Affiliations:** 1 European Centre for Disease Prevention and Control (ECDC), Stockholm, Sweden

In the article ‘Mid-season real-time estimates of seasonal influenza vaccine effectiveness in persons 65 years and older in register-based surveillance, Stockholm County, Sweden, and Finland, January 2017’ by M Hergens et al., published on 23 February 2017, the x-axes in Figure 2 were mistakenly marked 2015/16 instead of 2016/17. This mistake was corrected on 1 March 2017 on request of the authors.

